# Who Are You More Likely to Help? The Effects of Expected Outcomes and Regulatory Focus on Prosocial Performance

**DOI:** 10.1371/journal.pone.0165717

**Published:** 2016-11-08

**Authors:** Fengqiu Xiao, Zhiwei Zheng, Heyi Zhang, Ziqiang Xin, Yinghe Chen, Yiwei Li

**Affiliations:** 1 Institute of Developmental Psychology, School of Psychology, Beijing Normal University, Beijing, China; 2 China National Children’s Center, Beijing, China; 3 Key Laboratory of Mental Health, Institute of Psychology, Chinese Academy of Sciences, Beijing, China; 4 Psychology and Education Group, Faculty of Education, University of Cambridge, Cambridge, United Kingdom; 5 Department of Psychology at School of Social Development, Central University of Finance and Economics, Beijing, China; University of Würzburg, GERMANY

## Abstract

Prosocial behavior refers to a broad category of actions that benefit other people or the society. Compared with other factors that affect prosocial performance, prosocial outcomes, consisting of prosocial gains and prosocial non-losses have received less attention up to now. In the current research, we explored the influences of different types of expected outcomes and regulatory focus on prosocial performance. Studies 1a and 1b examined the differences in prosocial performance elicited by prosocial gain (e.g., enhancing others’ access to clean water) and prosocial non-loss outcomes (e.g., protecting others from suffering dirty water). We found that the expected prosocial non-loss outcomes induced greater prosocial performance compared with the expected prosocial gain outcomes. Studies 2a and 2b examined the effects of dispositional and situational regulatory focus on prosocial loss aversion. We found that differences in prosocial performance between two expected prosocial outcomes were reduced when promotion focus was primed; whereas a primed prevention focus did not significantly increase this difference. Additionally, participants displayed a greater prosocial loss aversion in the prevention focus condition than in the promotion focus condition. The reason for the non-significant interaction between regulatory focus and expected prosocial outcome was discussed.

## Introduction

Prosocial behavior refers to a broad category of actions that benefit other people or the society that we lives in, such as helping, comforting, sharing, cooperation, philanthropy, and community service [1]. A wide range of factors, including individual differences, situational variables, and outcome-related variables of prosocial behavior, have been found to influence prosocial behavior. Previous research has primarily focused on either the demographic and individual characteristics of the helper [1–3] or situational factors [4–6]; less attention has been paid to the roles of outcome-related variables.

A number of theories and studies have suggested that the expected outcomes influence individual motivation and behavior [7]. For example, the theory of planned behavior (TPB) states that personal evaluations, perceived social pressure, and perceived control should be considered as predictors of the intention to perform a given behavior [8]. Later, researchers developed the standard TPB model by including anticipated affective consequences (i.e., anticipated regret and donation anxiety) in predicting behavioral intentions [9]. And anticipating affective consequences was found to play an important role in the formation of intentions to donate blood [10]. Other studies also showed that appeals for donation could differ due to their presentation format [11].

However, most theories and studies only addressed the role of expected outcome in term of the helper. Little research has investigated the role of expected outcome relative to the beneficiary in predicting prosocial intention and behavior. In general, there are two types of expected outcomes for the beneficiary, obtaining positive outcomes and avoiding negative outcomes. Although both expected outcomes have equal positive valence for the beneficiary, their effects on helping willingness and behavior may be different. This hypothesis is derived from the theory and studies about loss aversion.

### Loss Aversion and Prosocial Outcomes

Loss aversion, which was originally proposed by Kahneman and Tversky in their prospect theory [12], demonstrates that negative events are more intense in terms of their objective magnitude than positive events [13]. In other words, people tend to weigh losses more heavily than gains with the same magnitude.

Subsequently, researchers have distinguished and compared four types of outcomes that include losses, gains, non-losses, and non-gains [14–17]. Losses and non-gains represent negative valence, whereas gains and non-losses represent positive valence. Previous studies on loss aversion have either primarily focused on the comparison between losses and gains [13,18–21] or the two types of negative valence [15–17,22,23]. The comparison between the two types of positive valences has received little attention. Tversky indicated that, in negotiations, eliminating losses (i.e., non-losses) were more effective than increasing gains [24]. Following this perspective, some researchers have hypothesized that, when the principle of loss aversion applies to positive valences in the same manner as it applies to negative valences, non-losses should be evaluated as more positive than gains [17]. Briefly, people are more likely to weigh non-losses heavily than gains with the same magnitude. However, this hypothesis was not supported when people made decision for themselves. How about when people make decision on behalf of others?

Numerous studies have discovered evidence of loss aversion, suggesting that loss aversion reflects a long-held, fundamental phenomenon [22,25]. These studies have primarily focused on risky decisions in the economic domain. However, much less is known about loss aversion in other areas. The present study aimed to examine whether the phenomenon of loss aversion occurs in the domain of prosocial behavior.

With regard to prosocial behavior, as discussed above, two types of outcomes need to be distinguished: attaining positive outcomes and avoiding negative outcomes. Combining the two types of prosocial outcomes and the relevant concepts of loss aversion, prosocial gain is defined as a situation, in which helper A performs prosocial behavior in order to attain positive outcome for beneficiary B; prosocial non-loss is defined as a situation, in which helper A performs prosocial behavior in order to avoid negative outcome for beneficiary B. Based on the rule of loss aversion, the first hypothesis for the present study is that prosocial non-loss outcome would induce more help than prosocial gain outcome, which is defined as prosocial loss aversion.

Based on the above discussion, the current study examined the phenomenon of prosocial loss aversion and explored its motivational mechanism. The motivational mechanism was addressed from the perspective of regulatory focus theory, examining the moderating effect of regulatory focus on the relationship between expected prosocial outcomes and prosocial performance.

### Regulatory Focus

Regulatory focus theory proposes that regulatory focus is a principle of self-regulation, which interprets the motivational differences of behaviors. People’s regulatory focus can be categorized into two subsets of motivational orientation. The promotion focus emphasizes nurturance and reward, and the prevention focus emphasizes security and safety [14]. The theory also suggests that people with distinct regulatory focus differ in their sensitivities to gains and losses. A promotion focus involves sensitivity to the presence and absence of positive outcomes. In contrast, the prevention focus is characterized by sensitivity to the presence and absence of negative outcomes [26–28]. Theoretically, people who are prevention focused are more likely to exhibit greater prosocial loss aversion than those with promotion focus. Empirical evidence supported the links between the two types of regulatory focus and the levels of sensitivity to losses and gains. For example, Idson et al. found that losing was experienced more intensely by participants in a state of prevention focus than those in a state of promotion focus, while the opposite was found in the case of winning [15].

Although the above studies indicate that individuals’ promotion and prevention focus are related to different levels of sensitivity to losses and gains respectively, no prior research has examined how regulatory focus may influence loss aversion in prosocial contexts. Prosocial behavior is different from behavior motivated by self-interests, referring to action that benefits others. Both gains and non-losses directed at others are prosocial outcomes. Polman suggested that loss aversion was reduced when promotion-focused people made choices for others compared to making choices for themselves, whereas prevention-focused people showed the same loss aversion in both circumstances [19]. The study of Polman suggested that the effect of regulatory focus on loss aversion directed at others might be different. A clear distinction has to be made between prosocial loss aversion and helpers’ regulatory focus, as the former is beneficiary-centered but the latter is helper-centered. Therefore, the second aim of the present study was to investigate the effect of the helpers’ regulatory focus on prosocial loss aversion.

### The Present Research

The present research consists of two studies. In Study 1, we investigated the differences of prosocial performances in helping others between attaining positive outcomes (defined as prosocial gains, e.g., enhancing other’s access to clean water) and avoiding negative outcomes (defined as prosocial non-losses, e.g., protecting other from suffering from dirty water). We hypothesized that prosocial non-loss outcomes would induce higher levels of prosocial performance compared with prosocial gain outcomes (defined as prosocial loss aversion). Unlike existing work that merely focused on the effect of expected outcomes on prosocial behavior from the perspective of helpers [9,10], the present research aimed to examine the effects of two types of expected outcomes on prosocial performance from the perspective of beneficiaries. In addition, the present research investigated the effect of expected prosocial outcome not only in terms of prosocial willingness but also prosocial behavior.

In Study 2, we shed light on the moderating effect of the helpers’ regulatory focus on the relationship between the expected prosocial outcome and prosocial performance. Based upon the theories and research about loss aversion and regulatory focus, we expected that promotion-focused individuals should help more when they anticipate gain as an outcome of their behavior; whereas prevention-focused individuals should help more when they anticipate non-loss as an outcome of their behavior.

Although several studies have examined individual differences and situational factors, scant attention has been paid to the factors related to prosocial outcomes and possible interactions between the different factors. Therefore, examining the interaction between regulatory focus and expected prosocial outcomes in the current work allowed us not only to explore the motivational mechanism of prosocial loss aversion, but also to examine the interaction between individual differences and outcome-related factors on prosocial performance. Furthermore, a moderation by dispositional and situational regulatory focus was examined.

## Study 1

In Study 1, we designed two mini-studies to investigate the phenomenon of prosocial loss aversion. In Study 1a, the participants were instructed to decide whether they preferred to help others attain positive outcomes or avoid negative outcomes. In Study 1b, participants were asked not only to decide whom they were more likely to help but also to rate the degree of willingness to help for each of the two expected prosocial outcomes. We expected that participants would display greater prosocial performance when they were asked to help others avoid negative outcomes than attain positive outcomes. The present study was approved by the ethical committee of the School of Psychology, Beijing Normal University. Participants provided written informed consent before the study.

### Study 1a

Study 1a provided an initial investigation of the phenomenon of prosocial loss aversion. A survey was used to investigate individuals’ preferences of expected prosocial outcomes.

#### Materials and methods

Participants: Hundred-and-six sophomores (73 females and 33 males) of China Youth University of Political Studies in Beijing completed a survey. These students participated in the study for credit toward a course requirement.

Procedure: All participants were provided with a sheet that indicated two victims were in a stricken area and had the same demands. The participants were told that they had the ability and opportunity to help victim A attain positive outcomes (e.g., enhancing victim A’s access to clean water) and to help victim B avoid negative outcomes (e.g., protecting victim B from suffering from dirty water). Then, they were asked which victim they would be more likely to help. The answer (help A or B) was recorded. The order of the presentation of the two expected prosocial outcomes was counterbalanced. The detailed scenario can be seen in S1 File.

#### Results and discussion

A chi-square test was conducted to examine the differences in the number of participants choosing for two prosocial outcomes. The result showed that participants preferred the prosocial non-loss outcome (*N* = 67) compared to prosocial gain outcome (*N* = 39), *χ*^2^ (1) = 7.40, *p* = .007, suggesting a greater tendency toward prosocial non-losses than prosocial gains.

This result suggests individuals tend to help others avoid negative outcomes rather than to attain positive outcomes. Given that this survey only investigated the preference for expected prosocial outcomes, Study 1b was performed to further examine the differences between the degrees of individuals’ willingness to help for prosocial gain and prosocial non-loss outcomes.

### Study 1b

In Study 1b, both the preference for and the exact degrees of prosocial willingness for prosocial gain and prosocial non-loss outcomes were measured and compared.

#### Materials and methods

Participants: The participants were an additional sample of 60 sophomore students (38 females and 22 males) recruited from China Youth University of Political Studies in Beijing, China. They participated in the experiment for credit of a psychology course.

**Fig 1:**
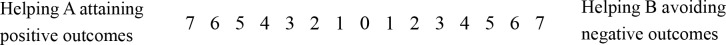
The scale of helping victim A attain positive outcomes and helping victim B avoid negative outcomes.

Procedure: The procedure and materials provided to the participants were similar to those used in Study 1a with the exception that participants were also required to indicate the degree to which they wanted to help for each of the two expected prosocial outcomes on a bipolar scale (see Fig 1). The larger number indicated that the participants would be more likely to help, and the victims would get more help. The positions of the two expected prosocial outcomes on the bipolar scale were counterbalanced. The detailed scenario can be seen in S1 File.

#### Results and discussion

A chi-square test revealed that the difference between the number of each of two choices was statistically significant, *χ*^2^ (1) = 4.27, *p* = .039. Thirty-eight participants indicated that they would like to help the victim avoid negative outcomes, and 22 participants indicated that they would like to help the victim attain positive outcomes.

To further examine the difference in the degree of prosocial preference, a paired-samples *t*-test was performed on the willingness to help for the prosocial gain and non-loss outcomes. Among the 60 participants in the experiment, 55 participants responded to both outcomes. The results revealed that the participants expressed greater willingness to help the victim avoid negative outcomes (*M* = 5.73, *SD* = 1.50) than attain positive outcomes (*M* = 5.09, *SD* = 1.49), *t* (54) = 2.38, *p* = .021, *d*_*z*_ = 0.32.

These findings suggested that prosocial non-losses evoked greater prosocial willingness than prosocial gains did. In other words, loss aversion occurred in the prosocial domain, which is in line with previous research results [11].

Although the main findings were in line with our predictions, one potential limitation was that both Studies 1a and 1b were conducted in a classroom context, and the situation described in the materials was unfamiliar to the participants. To address the limitation, prosocial performance measured in Study 2 was conducted in a more realistic prosocial situation. In addition, the present order of two expected prosocial outcomes was only controlled, but the effect was not reported in Study 1. This order effect would be tested in Study 2.

## Study 2

Although differences were found between participants helping others avoid negative outcomes and helping others attain positive outcomes, the reasons for these differences have not been fully understood. Despite a growing body of research focusing on the motivational mechanism of prosocial behavior [4,29–31], little research, if any, has examined motivational differences between the two types of prosocial outcomes. To fill this gap, Study 2 aimed to explore whether the differences in prosocial performances between prosocial gain and prosocial non-loss outcomes can be explained by regulatory focus theory.

According to the regulatory focus theory and related research [26, 32], we hypothesized that potential helpers with a prevention focus would be more likely to help others avoid negative outcomes than help others achieve positive outcomes. This would magnify the degree of loss aversion. In contrast, it is assumed that potential helpers with a promotion focus would be more prone to help others achieve positive outcomes than avoid negative outcomes, which might cause a reduction or even a reversal of loss aversion.

In Study 2, the focus on promotion versus prevention was manipulated either as a dispositional individual difference or a situational difference by priming [33,34]. Two mini-studies were designed to examine the moderating effects of regulatory focus. In Study 2a, the regulatory focus at the dispositional level was measured by the regulatory focus scale. The goal of Study 2a was to investigate individual differences in prosocial loss aversion in terms of dispositional regulatory focus, and to preliminarily examine the moderating effect of regulatory focus on the relationship between expected prosocial outcome and prosocial behavior. In Study 2b, the regulatory focus at the situational level was primed by helping a mouse out of a maze. The goal of Study 2b was to investigate the motivation of prosocial loss aversion by directly testing the moderating effect of situational regulatory focus.

Meanwhile, to address the limitation about material, prosocial performance measured in Study 2 was conducted in a more realistic prosocial situation. The fictitious victims were not sufferers in a stricken area but were similarly aged peers of the participants. The prosocial gains were helping a stranger attain money, and the prosocial non-losses were helping a stranger avoid losing money. The participants were instructed to indicate that the extent to which they were willing to help for each of the two expected prosocial outcomes. Furthermore, two qualifying tests measured the participants’ real prosocial behaviors for each of the two expected prosocial outcomes. The participants were told that they would be able to help the strangers in need only on the condition of passing the qualifying test. The underlying logic of the qualifying test was that if the participants truly wanted to help, they would do their best to pass the qualifying test, thereby performing at a high level [35]. Thus, the performances in two qualifying tests could be used as indices of their prosocial behaviors for the two expected prosocial outcomes. Additionally, the difference between their performances in two qualifying tests served as an additional index of their prosocial loss aversion.

### Study 2a

To indirectly explore the motivational mechanism that underlies the occurrence of prosocial loss aversion, Study 2a examined the interaction effect between individual differences and outcome-related factors on prosocial performance from the perspective of the dispositional regulatory focus.

#### Materials and methods

Participants: Sixty-two undergraduate students (45 males and 17 females, *M*_age_ = 20.68, *SD* = 1.08) were recruited from Tianjin Chengjian University in China. Each participant received a gift for participating after the experiment. The experiment was approved by the Ethical Committee of the School of Psychology, Beijing Normal University. Consent forms were given and signed by the participants prior to the experiment.

Procedure and Measures: Participants were randomly assigned to six groups with 10–12 members. Upon arriving at the laboratory, the participants were asked to independently complete a battery of paper-pencil questionnaires concerning regulatory focus and prosocial loss aversion for prosocial willingness and behavior.

*Dispositional regulatory focus*. The Regulatory Focus Scale (RFS, 33-items, [36]) was used to measure dispositional regulatory focus. The participants rated the items on a scale from 1 (*definitely untrue for me*) to 7 (*definitely true for me*). The RFS is divided into two subscales: Promotion focus (17 items) and Prevention focus (16 items). The promotion subscale (Cronbach’s *α* = .67) consists of statements that reflect a focus on achieving positive things (e.g., “It is very important to me to develop myself further and to improve myself”). The prevention subscale (Cronbach’s *α* = .78) consists of statements that reflect a focus on avoiding negative things (e.g., “I often think about how I can avoid failures in my life”). This scale has been shown to be reliable and valid in previous research examining individual regulatory focus orientations [36–38].

*Prosocial loss aversion for prosocial willingness and behavior*. The participants were provided with a sheet that contained information about a prosocial scenario. In the scenario, two strangers had not received all of the money owed them from another experiment because they failed to complete all of the experimental trials. If participants wanted to help the strangers, they needed to complete trials for the strangers. The independent variable was two types of expected prosocial outcomes: prosocial gains (i.e., helping a stranger attain the remaining money) and prosocial non-losses (i.e., helping a stranger avoid losing money they had already received due to the uncompleted trials).

First, participants were asked to choose the number of trials, from 0 to 10, that they would be willing to complete for each of the two strangers separately. After finishing these choices, the participants were told that they would have the opportunity to help the strangers only if they passed the qualifying tests. If the participants truly wanted to help, they would do their best in the qualifying tests, and their performances in these tests would represent their prosocial behavior.

The qualifying tests were adapted from the Number Cancellation and Letter Cancellation tests which were used to measure selective attentional abilities [39–41]. The Number Cancellation Test was one page in length with 25 rows and 40 numbers (from 0 to 9) in each row. The Letter Cancellation Test was one page in length with 25 rows and 40 letters (from A to I) in each row. All the numbers or the letters were randomly interspersed. The participants were instructed to cross out all the target numbers and letters respectively. Specifically, for the target number, the participants were asked to choose the number between 3 and 7 or 7 and 3. For the target letter, the participants were asked to choose the letter between D and H or H and D. The target numbers and target letters were equal in number. Participants had 4 minutes for two tests and had the freedom to allocate time to each test. Each qualifying test corresponded to one type of expected prosocial outcomes.

For half of the participants, the qualifying test under the condition of the prosocial gain outcome was the Number Cancellation Test; the qualifying test under the condition of the prosocial non-loss outcome was the Letter Cancellation Test. While for the other half of the participants, the qualifying test under the condition of the prosocial gain outcome was the Letter Cancellation Test; the qualifying test under the condition of the prosocial non-loss outcome was the Number Cancellation Test. The order and correspondence of the two qualifying tests with two expected prosocial outcomes were counterbalanced. The dependent measures were the numbers of the trials selected for two expected prosocial outcomes and the performances in the two cancellation tests. After completing the experiments, the participants were thanked and debriefed by the experimenter. When asked, none of them reported any hypotheses relevant to the true purpose of the experiment. The detailed scenario can be seen in S1 File.

#### Results and discussion

Basic descriptive data on regulatory focus, prosocial willingness, and prosocial behavior is presented in Table 1.

**Table 1:** Descriptive data on regulatory focus, prosocial willingness, and prosocial behavior.

Variable	*Min*	*Max*	*M*	*SD*
Promotion focus	3.00	6.00	4.93	0.61
Prevention focus	2.94	6.50	5.19	0.71
Prosocial gain willingness	0	10	4.24	3.21
Prosocial non-loss willingness	0	10	5.46	3.26
Prosocial gain behavior	0	25	6.97	7.54
Prosocial non-loss behavior	0	25	12.62	8.48

Two independent *t* tests of prosocial loss aversion on order and counterbalance were conducted. The effects of order (*p* = .756) and counterbalance (*p* = .095) were not significant.

Two hierarchical regression analyses of prosocial willingness on expected prosocial outcome (prosocial gains vs. prosocial non-losses) and regulatory focus (promotion vs. prevention) were conducted. The type of expected prosocial outcomes was coded into a dummy variable (“0” for prosocial gains and “1” for prosocial non-losses). The continuous measures of promotion and prevention were centered prior to computing the interactions and used as the main effects respectively. Model 1 tested the effects of promotion focus, the type of expected prosocial outcomes, and their interaction on prosocial willingness. In the hierarchical regression, centered prevention focus as a controlled variable was entered in the first step, centered promotion focus and the type of expected prosocial outcomes were included in the second step, and the interaction between centered promotion focus and the type of expected prosocial outcomes were included in the third step.

Model 2 tested the effects of prevention focus, the type of expected prosocial outcomes, and their interaction on prosocial willingness. In the hierarchical regression, centered promotion focus as a controlled variable was entered in the first step, centered prevention focus and the type of expected prosocial outcomes were included in the second step, and the interaction between centered prevention focus and the type of expected prosocial outcomes were included in the third step. The analyses revealed two main effects of the types of expected prosocial outcome. No significant main effect of promotion focus, prevention focus, or interaction was found (see Table 2).

**Table 2:** The effects of expected prosocial outcome, promotion focus and prevention focus on prosocial willingness.

*Model*		*β*	*T*	*p*
Model 1	Prevention focus (Pre)	.104	1.124	.264
	Type of Expected prosocial outcome (EPO)	.184	1.980	.050
	Promotion focus (Pro)	.009	.066	.948
	EPO×Pro	.082	.631	.529
Model 2				
	Promotion focus (Pro)	.066	.711	.478
	Type of Expected prosocial outcome (EPO)	.191	2.050	.043
	Prevention focus (Pre)	.151	1.151	.252
	EPO×Pre	-.066	-.507	.613

Two similar separated hierarchical regression analyses concerning prosocial behavior were conducted. Similarly, regressions on promotion focus and on prevention focus were conducted separately. The results revealed two main effects of expected prosocial outcomes. No significant main effect of promotion focus, prevention focus, or interaction was found. No other significant effect was found (see Table 3).

**Table 3:** The effects of expected prosocial outcome, promotion focus and prevention focus on prosocial behavior.

		*β*	*T*	*p*
Model 1	Prevention focus (Pre)	-.004	-.045	.964
	Type of Expected prosocial outcome (EPO)	.334	3.804	.000
	Promotion focus (Pro)	.062	.496	.620
	EPO×Pro	-.019	-.149	.882
Model 2				
	Promotion focus (Pro)	.049	.549	.584
	Type of Expected prosocial outcome (EPO)	.335	3.810	.000
	Prevention focus (Pre)	-.002	-.016	.987
	EPO×Pre	-.003	-.023	.982

Because dummy variable 1 represented prosocial non-losses, the main effects of type of expected prosocial outcome indicated that the more prosocial non-losses, the more prosocial willingness and behavior.

Therefore, both analyses of prosocial willingness and behavior exhibited a significant main effect of expected prosocial outcome, indicating that the prosocial performances for the prosocial non-loss outcome were greater than those for the prosocial gain outcome. Furthermore, this experiment indicated that only the expected prosocial outcome affected prosocial willingness and prosocial behavior, whereas individuals’ dispositional regulatory focus did not affect the relationship between expected prosocial outcome and prosocial performance, which was inconsistent with our hypothesis. The non-significant moderating effect of dispositional regulatory focus may due to the non-significant difference between dispositional promotion and prevention focuses. The sample size was not large enough to distinguish between participants who reported high and low promotion and prevention focus orientations, respectively.

Study 2a made a primary examination of the moderated effect of dispositional regulatory focus. Study 2b distinguished two regulatory focuses by situational priming and tested the hypothesis about the moderating effect of situational regulatory focus.

### Study 2b

Following Study 2a, in order to directly identify the motivational mechanism that underlies the occurrence of prosocial loss aversion, Study 2b manipulated situational regulatory focus via experimental priming and examined the interaction between situational regulatory focus and expected prosocial outcomes.

#### Materials and methods

Participants: One hundred and six undergraduates (65 males; *M*_age_ = 20.86, *SD* = 1.36) from Tianjin Chengjian University in China participated in this experiment. The participants were compensated with a gift for their participation. The present experiment was approved by the Ethical Committee of the School of Psychology, Beijing Normal University. Consent forms were presented and signed by the participants before the experiment.

Procedure and Measures: Upon arriving at the laboratory, the participants were instructed to independently complete a series of ostensibly unrelated essay tasks. The participants were randomly assigned into three conditions. Two groups were assigned to one pair of mazes that have previously been demonstrated to elicit either a promotion or prevention focus [33,42]. In the promotion-focused-priming condition, the participants were instructed to help the mouse in the maze move toward the cheese. In the prevention-focused-priming condition, the participants were instructed to help the mouse in the maze escape from a hawk. After completing one of the mazes, each participant received the sheet used in Study 2a to measure their prosocial willingness and behavior. The participants in non-priming condition only finished the measure of prosocial willingness and behavior.

#### Results and discussion

The participants’ responses regarding prosocial willingness were submitted to a 2 (expected prosocial outcome: prosocial gains vs. prosocial non-losses) × 3 (regulatory focus: promotion vs. prevention vs. non-priming) mixed factorial design ANOVA, which yielded no significant effects, *F*s < 1.71, *p*s > .05.

An analogous 2 × 3 repeated measures ANOVA on prosocial behavior revealed a significant main effect of expected prosocial outcome (*F* (1, 103) = 14.93, *p* < .001, *η*^2^ = .13) and no significant main effect of regulatory focus (*p* = .251). The interaction between expected prosocial outcome and regulatory focus was not significant, *p* = .063. Based on the strong hypotheses, simple effect analyses were conducted and revealed that participants whose prevention focus was primed showed a better performance for prosocial non-loss outcome than prosocial gain outcome. Similarly, participants in the non-priming condition also performed better for the prosocial non-loss outcome. However, no significant difference was found between the performances of prosocial non-loss and gain outcomes for participants whose promotion focus was primed. These results are presented in Table 4. Meanwhile, the prosocial loss aversion in the promotion-focus-priming condition was significantly lower than those in the prevention-focus-priming and non-priming conditions. And the difference in prosocial loss aversion between the prevention-focus-priming and non-priming conditions was insignificant.

**Table 4:** Prosocial performance as a function of situational regulatory focus and expected prosocial outcome.

Regulatory focus	Expected prosocial outcome	*M*	*SD*	*F*	*df*	*p*
Promotion focus	Prosocial gain outcome	9.20	9.49	0.09	1	.798
	Prosocial non-loss outcome	9.89	7.91			
Prevention focus	Prosocial gain outcome	4.64	5.54	9.64	1	.002
	Prosocial non-loss outcome	11.54	7.40			
Non-priming	Prosocial gain outcome	4.63	7.26	10.89	1	.003
	Prosocial non-loss outcome	12.72	9.33			

The findings of Study 2b only revealed that participants in the promotion-focus-priming condition tend to display lower prosocial loss aversion than that in prevention-focus-priming condition. A lack of significant interaction between expected prosocial outcome and situational regulatory focus might due to the weak priming manipulation of regulatory focus, especially for prevention focus. Another reason for the insignificant interaction might be the small sample size.

## General Discussion

The purpose of the present research was to examine the effect of expected prosocial outcomes on prosocial performance. Four experiments provided evidence that people were more likely to help others avoid negative outcomes than attain positive outcomes, which supported the occurrence of prosocial loss aversion. Additionally, the interaction effect of expected prosocial outcomes and regulatory focus on prosocial performance was examined. We only found that the prosocial loss aversion in the promotion-focus-priming condition was significantly lower than in the prevention-focus-priming and non-priming conditions. And the difference in prosocial loss aversion between the prevention-focus-priming condition and non-priming conditions was insignificant.

This finding that non-loss outcomes induced more help than gain outcomes is consistent with the principle of loss aversion that losses exert greater influences on choice and predicted feelings about an outcome than do gains of the same magnitude [25,43,44]. The current research shed light on loss aversion framed in the prosocial context in which prosocial loss aversion was reflected by the fact that prosocial non-losses induced greater prosocial performances than prosocial gains did.

However, strictly speaking, the outcomes of events can be classified into losses, gains, non-losses, and non-gains. As mentioned before, gains and non-losses represent positive valences, while losses and non-gains represent negative valences. In previous studies of loss aversion, particular attention has been paid to gain-loss comparisons. Losses have been found to exert twice as much influence on decisions as equivalent gains [44]. Kahneman and Miller suggested that the natural comparison should be a set of events with the same valence [45]. Therefore, it is important to distinguish gains from non-losses and losses from non-gains [14,15]. For example, Liberman et al. proposed that, according to the principle of loss aversion, losses should be perceived as more intensely negative than non-gains, while non-losses should be perceived as more positive than gains [17]. However, their studies confirmed the first prediction, but failed to corroborate the second one. The current study showed that an outcome that resulted in a non-loss induced more help than an outcome that resulted in a gain.

One reason for the discrepancies between the findings of the previous and the current research might be attributable to the differences in the experimental situations. Unlike the present research conducted in prosocial situations, previous studies were mainly conducted in the economic domain. Additionally, loss aversion examined in previous studies merely applied to the participants themselves, whereas in the present research, the decisions applied to others to some extent. Prosocial behavior refers to behavior that benefits other people or society. Both prosocial gains and prosocial non-losses incur benefits directed at other people. Although some studies have shown that loss aversion is reduced when people make choices for others, compare to making choices for themselves [19], others have reported that cognitive biases are stronger when decision makers choose on behalf of others than they choose on their own behalves [46, 47]. As decisions in prosocial scenarios are more likely to be made on behalf of others, prosocial scenarios induce significant prosocial loss aversion.

The other purpose of the present studies was to examine the interaction effect of expected prosocial outcome and regulatory focus on prosocial performance. The result partly supported the hypothesis: prosocial loss aversion in the promotion-focus-primed condition was significantly lower than in the prevention-focus-priming and non-priming condition. These findings are supported by the regulatory focus theory and the regulatory fit theory [14–17]. In addition, the present studies also showed that neither dispositional nor situational regulatory focus significantly interacted with expected prosocial outcome on prosocial performance. These results were not consistent with the study of Fransen et al. [48]. Their study reported that individuals in a state of prevention focus donated more money when the goals of a charity were described as preventing a negative outcome. And individuals in a state of promotion focus donated more money when the goals were presented as encouraging positive outcomes [48]. The inconsistent might due to the fact that the difference between promotion and prevention focus in the current study was not large enough. For the dispositional regulatory focus, the scores of participants’ promotion focus showed in Study 2a were similar to those of prevention focus. For the situational regulatory focus, the priming might be weak. Alternatively, the sample size in either study was not large enough.

Despite the valuable findings of the current research, several limitations should be noted. First, as mentioned before, the outcomes of events can be classified into losses, gains, non-losses, and non-gains. Further research on prosocial loss aversion is necessary to include both positive-valence helping outcomes (gains and non-losses) and negative-valence non-helping outcomes (losses and non-gains) to allow for a clearer examination. Second, although we consistently observed prosocial loss aversion across four experiments, more diverse prosocial domains with different risk should be included to examine the phenomenon of prosocial loss aversion in future studies. Third, more detailed information should be considered. For example, the present order of two expected prosocial outcomes in Study 1 should be recorded and analyzed. The balance between the speed and the accuracy of the cancellation task, and the relationship between dispositional regulatory focus and the balance should be considered in future study. Fourth, for the non-significant dispositional regulatory focus on prosocial loss aversion, future studies may include large enough sample size to distinguish apparent promotion and prevention focus individuals. For the non-significant situational regulatory focus on prosocial loss aversion, future studies may use stronger priming condition and add operational check after priming. Finally, the current study only focused on the effect of the beneficiaries’ outcomes. However, prosocial behavior has impacts on both helpers and beneficiaries. The expected outcome of helpers’ prosocial behavior also affected their prosocial performance [9]. The interaction between expected outcomes to helpers and beneficiaries and the relationship between helpers and beneficiaries (in- vs. out-group) is worthwhile considering in a follow-up study.

## Conclusions

The present studies explored how expected prosocial outcomes affected people’s prosocial performances. The findings indicated that the phenomenon of loss aversion also occurred in the prosocial domain, in which expected prosocial non-loss outcomes induced higher levels of prosocial performance than expected prosocial gain outcomes did. The present studies also showed that prosocial loss aversion in the prevention-focus-priming condition was significantly higher than in promotion-focus-priming and non-priming condition. Prosocial loss aversion might be lessened when promotion focus was primed, whereas prosocial loss aversion was not significantly increased when prevention focus was primed. The non-significant interaction between expected prosocial outcomes and regulatory focus might due to the shortcomings of the current studies.

## Supporting Information

S1 File The experimental materials for Studies 1a, 1b, and 2. (DOCX)

S2 File The codebook for variables in the data files. (DOCX)
