# Supplementary material for: Who Are You More Likely to Help? The Effects of Expected Outcomes and Regulatory Focus on Prosocial Performance
Source: PLoS One. 2016 Nov 8;11(11):e0165717. doi: 10.1371/journal.pone.0165717 (PMC5100932; doi:10.1371/journal.pone.0165717)
Supplement: S1 File — (DOCX) [file pone.0165717.s001.docx]

# Study 1a

Both A and B are children from disaster area, and they are under totally the same condition now. If you are able to help them, your help would:

**Let A obtain positive outcomes, such as drinking clear water, eating healthily, going to school if he needs, or reducing health risks.**

**Let B avoid negative outcomes, such as drinking dirty water, eating unhealthily, dropping school or increasing health risks (as malaria).**

Which child do you prefer to help, A or B (Please write down your choice ).

# Study 1b

Both A and B are children from disaster area, and they are under totally the same condition now. If you are able to help them, your help would:

**Let A obtain positive outcomes, such as drinking clear water, eating healthily, going to school if he needs, or reducing health risks.**

**Let B avoid negative outcomes, such as drinking dirty water, eating unhealthily, dropping school or increasing health risks (as malaria).**

Which child do you prefer to help, A or B (Please write down your choice ). And then choose from the numbers below to represent how willing you are willing to help him/her. **The bigger the number, the stronger your willingness is. Numbers to the left of 0 represent the degree of your willingness to help A, while numbers on the right to 0 represent the degree of your willingness to help B. You need to choose the degree of wiling for A and B separately and circle the numbers.**

| **Helping A to obtain positive outcomes** | 7 | 6 | 5 | 4 | 3 | 2 | 1 | **0** | 1 | 2 | 3 | 4 | 5 | 6 | 7 | **Helping B to avoid negative outcomes** |
| --- | --- | --- | --- | --- | --- | --- | --- | --- | --- | --- | --- | --- | --- | --- | --- | --- |

# Study 2

**Please read the following material carefully and follow the instructions, then make a judgment by yourself. This study is based on real life circumstances, and we are arranging the following experiment according to the result.**

Here are 2 experiments that are boring and time-consuming. Each experiment has 20 trials and each trial takes 10 minutes, which is, 200 minutes in total. Many participants didn’t finish the whole experiments. Participant M only finished 10 trials and was paid 30 yuan. **He can get another 30 yuan if you finish the rest of experiment for him.** Are you willing to continue the experiment in order to help M to get the rest 30 yuan? How many trials would you like to finish? Please make a choice.

| 0 | 1 | 2 | 3 | 4 | 5 | 6 | 7 | 8 | 9 | 10 |
| --- | --- | --- | --- | --- | --- | --- | --- | --- | --- | --- |

Participant N only finished 10 trails as well. Based on the completion status, N was also paid 30 yuan, **and another 30 yuan was suspended. He could receive those suspended 30 yuan if you finish the rest of experiment for him.** Are you willing to continue the experiment in order to help N to avoid being deducted 30 yuan? How many trials o you would you like to finish? Please make a choice.

| 0 | 1 | 2 | 3 | 4 | 5 | 6 | 7 | 8 | 9 | 10 |
| --- | --- | --- | --- | --- | --- | --- | --- | --- | --- | --- |

**If you want to help M to obtain the rest 30 yuan, you need to take qualification test 1. Only after you pass the test, you have the chance to continue the boring experiment and help M to get the rest 30 yuan. If you fail to pass the test, you cannot help M.**

**If you want to help N to avoiding being deducted 30 yuan, you need to take qualification test 2. Only after you pass the test, you have the chance to continue the boring experiment and help N to avoid being deducted 30 yuan. If you fail to pass the test, you cannot help N.**

In order to help subject M to get the rest 30 yuan, the qualification test is on the following page:

In order to help subject N to avoid being deducted 30 yuan, the qualification test is on the following page:

You have 4 minutes in total, which could be allocated for two qualification tests on your own. Please finish the test for the person who you prefer to help first.

**(The following sentences were presented at the top of two qualification tests respectively.)**

Remember only after you pass the test, you have the chance to continue the boring experiment and **help M to get the rest 30 yuan.** If you fail to pass the test, you cannot help M.

Remember only after you pass the test, you have the chance to continue the boring experiment and **help N to avoid being deducted 30 yuan.** If you fail to pass the test, you cannot help N.
