# Supplementary material for: Who Are You More Likely to Help? The Effects of Expected Outcomes and Regulatory Focus on Prosocial Performance
Source: PLoS One. 2016 Nov 8;11(11):e0165717. doi: 10.1371/journal.pone.0165717 (PMC5100932; doi:10.1371/journal.pone.0165717)
Supplement: S2 File — (DOCX) [file pone.0165717.s002.docx]

Codebook for variables

In Study 1a:

Choice:

A--- helping others attain positive outcomes;

B---helping others avoid negative outcomes.

In Study 2a:

Order:

1--- prosocial gains condition was presented first;

2--- prosocial non-losses condition was presented first.

Material:

1--- the Number Cancellation test was used in prosocial gains condition and the Letter Cancellation test was used in prosocial non-losses condition;

2--- the Number Cancellation test was used in prosocial non-losses condition and the Letter Cancellation test was used in prosocial gains condition;

In Study 2b

Regulatory focus

1---promotion-focus-priming condition;

2---prevention-focus-priming-condition;

3----non-priming condition.

The code for “letter/number” is the same as “material” in Study 2a.
